# The Pharmacological Window Hypothesis: How Emerging Obesity Medications May Realize Decades of Plant-Forward Dietary Recommendations^☆^

**DOI:** 10.1016/j.advnut.2026.100689

**Published:** 2026-06-19

**Authors:** Robert E Bergia, Andrew W Brown, Michelle M Braun, Mark J Messina

**Affiliations:** 1 Berkwood Research, Santa Barbara, CA, United States; 2 Arkansas Children’s Research Institute, Little Rock, AR, United States; 3 University of Arkansas for Medical Sciences, Department of Biostatistics, Little Rock, AR, United States; 4 Soy Nutrition Institute Global, Washington, DC, United States

**Keywords:** GLP-1 receptor agonists, incretin mimetics, dietary patterns, food noise, dietary guidelines, weight management, behavior change

## Abstract

For decades, dietary guidance has been a signal lost in the noise of our modern food environment, resulting in stagnant indices of diet quality despite clear dietary recommendations. Emerging obesity medications (EOMs), including glucagon-like peptide-1–based therapies, appear to quiet-intrusive, cue-driven food noise and recalibrate reward responsivity to energy-dense foods. We propose that this pharmacological dampening of automatic motivation creates a transient “pharmacological window” in which the enduring elements of dietary guidance—emphasis on fruits, vegetables, legumes, whole grains, nuts, seeds, and limits on added sugars, sodium, and saturated fat—may be adopted with less friction than ever before. Synthesizing observational and experimental evidence, we describe the convergent effects of EOMs on ad libitum intake, selective reductions in wanting for high-fat/high-sugar foods with relative sparing for fruits and vegetables, and late-meal shifts that further favor plant-forward choices. Leveraging this window, we outline a pragmatic, identity-congruent approach to habituate plant-forward routines aimed at long-term health and sustainability benefits. Given the high discontinuation rates and likely therapy fluctuation, we argue that success in this era should not be judged only by weight loss, but by the durable, value-aligned habits that persist long after. Rather than supplanting nutrition guidance, EOMs may render it newly tractable by turning down the noise that has historically overwhelmed adherence.

**Statement of Significance**

This Perspective proposes that emerging obesity medications create a transient pharmacological window in which long-standing, plant-forward elements of dietary guidance may be adopted with less friction. Operationalizing this window may yield durable, identity-anchored habits that persist beyond pharmacotherapy.

## Introduction

Although controversies within the field of nutrition are commonplace, there is considerably more agreement than disagreement about the elements of a healthful diet [[1], [2], [3]]. Key recommendations, such as those included in the Dietary Guidelines for Americans (DGAs), have remained remarkably consistent from 1980 through 2025 (although the 2025–2030 DGAs explicitly departed from several recommendations [4] within the Dietary Guidelines Advisory Committee scientific report), emphasizing a focus on whole foods like legumes, nuts, seeds, whole grains, fruits, and vegetables and limits on added sugar, sodium, and saturated fat [5]. Despite this long-standing consensus, the dietary habits of Americans have improved little over the past several decades and remain quite discordant from recommendations [1,6]. Awareness and use of the DGAs and related meal patterning materials (such as MyPlate) remain limited; in 2024 only 32.5% of Americans reported knowing about MyPlate and only about half of those (14.8%) reported knowing about and trying to follow these recommendations [7].

These gaps between intake and recommendations persist despite decades of public health efforts. Consider the case of fruits and vegetables. The 5-a-day campaign launched in the early 1990s sought to raise intake to ≥5 daily servings [8], a target supported by a meta-analysis of 26 prospective studies indicating that mortality risk reduction plateaus at ≈5 servings of fruit and vegetables per day [9]. Yet mean daily United States intake remains at only 0.72 and 1.60 servings of whole/intact fruit and vegetables, respectively [10]. Similarly, fiber intake is at only approximately half of the recommendations [10], and pulses are consumed at a mean of only 0.39 oz/d [11]. Despite the reported benefits, pulse intake is associated with lower socioeconomic status and hold poor consumer perceptions [11].

These intake shortfalls are unlikely to be explained by knowledge gaps alone. As the 2010 DGA Committee observed, “…mere repetition of advice will not effectively help Americans achieve these evidence-based and often repeated goals for a healthy diet” [12]. Decades of education, advocacy campaigns, and behavioral nudging have yielded diminishing returns, suggesting that the key barriers may lie beyond awareness. The implication is not that dietary guidance has failed; it is that dietary guidance alone is unlikely to overcome the barriers inherent to a modern environment [13].

One barrier is “food noise,” recently defined as “persistent thoughts about food that are perceived by the individual as being unwanted and/or dysphoric and may cause harm to the individual, including social, mental, or physical problems” [14,15]. Food noise is distinguished from routine food-related thoughts by its intensity and intrusiveness, resembling rumination. Discussion of food noise has risen in prominence because a new generation of therapies—hereafter referred to collectively as emerging obesity medications (EOMs; also referred to as antiobesity medications) (Box 1 [16])—often seems to abruptly quiet it [17]. Because they quiet food noise, EOMs may create a window in which more individuals can focus on healthful eating instead of being preoccupied with thoughts about foods often of poor nutrient density. With less immediate hedonic pressure, these individuals may also be more receptive to dietary counseling, elevating the role of registered dietitian nutritionists (RDNs).

BOX 1 **A Primer on Emerging Obesity Medications**

The term emerging obesity medications refers to the rapidly evolving class of incretin-based therapies approved for weight management. These drugs mimic the action of natural gut hormones (albeit at supraphysiological levels and greatly extended half-lives). The foundational agents are glucagon-like peptide-1 (GLP-1) receptor agonists, such as liraglutide and semaglutide. More recent and potent therapies include dual-agonists, targeting both the GLP-1 and glucose-dependent insulinotropic polypeptide receptors, with *tirzepatide* being the first in this class. Numerous other variants and modalities are under development. Although their specific mechanisms and weight-loss efficacy vary, this paper discusses them collectively for their shared and profound effects on homeostatic and hedonic control of eating, particularly their ability to reduce food noise. Important pharmacologic heterogeneity nonetheless exists across this class. Magnitude of weight loss, modulation of cue-reactivity, and numerous other considerations may vary across single-agonists (e.g., liraglutide, semaglutide), dual-agonists (e.g., tirzepatide), and the rapidly expanding pipeline of triagonists, oral formulations, and combination therapies (e.g., orforglipron, retatrutide) [16].

Alt-text: BOX 1

This new environment is particularly timely because of the urgency of moving toward plant-forward dietary patterns. For both environmental [3,18] and health [[19], [20], [21]] reasons, high-income nations are being urged to adopt more plant-forward dietary patterns through the replacement of some animal protein with sources of plant protein. All 3 example dietary patterns in the 2020–2025 DGA—the Healthy United States–style, Healthy Mediterranean–style, and Healthy Vegetarian patterns—emphasize plant foods considerably more than the current United States dietary pattern [1]. Even the 2025–2030 DGA, which encourages greater consumption of both animal and plant sources of protein, places substantial emphasis on plant-based foods [22]. In high-income countries, including the United States, the dietary plant-to-animal protein ratio is ∼1:2 [23]. There is no agreed-upon optimal ratio, but moving from the current ratio to a pragmatic 1:1 ratio, wherein protein is provided equally by plant and animal sources, is likely to be an achievable and beneficial shift [24]. In support, a recent joint advisory on nutritional priorities to support glucagon-like peptide-1 therapy recommends an emphasis on diverse fruits, vegetables, legumes, whole grains, lean proteins, nuts, and seeds, with avoidance of red and processed meats [25]—recommendations that align with a plant-forward dietary approach.

Although consensus on the key elements of a healthful diet is not new, the opportunity to finally realize adherence to it may be. EOMs stand to create such an opportunity by opening a window in which potent barriers to healthful diets—such as automatic, cue-driven wanting for energy-dense foods—are mitigated. Recently, it was proposed that EOM-induced mitigation of the overwhelming “noise” of energy balance permits nutrition’s “signals” to emerge and be measured for what they are: potent levers on more nuanced endpoints such as blood pressure, LDL cholesterol, glycemia, and muscle health that do not depend on indefinite, purposeful caloric restraint [26]. This shift parallels the reappraisal of physical activity, whose most evident benefits appear to be independent of weight loss [27,28]. By quieting the food noise [14] and altering reward responsivity [29,30], EOMs may rebalance choice in favor of options that align with long-standing dietary guidance. The novelty is not in the target pattern; it is in the tractability in achieving a target pattern.

In this Perspective, we explore how EOMs might create a “pharmacological window” in which long-standing plant-forward dietary guidance can be translated into durable routines. In the following sections we describe how quieting the food noise may facilitate a new dietary paradigm, and how the pharmacological window could enable a plant-forward eating pattern to be habituated thereby promoting more durable weight management. We frame this argument through the lens of the Capability, Opportunity, Motivation-Behavior (COM-B) model, which posits that behavior requires the convergence of capability, opportunity, and motivation [31]. Motivation comprises both reflective components (conscious goals, values, and identity) and automatic components (impulses, habits, cravings, and wanting). We propose that EOMs may primarily influence the balance between automatic and reflective motivation. By attenuating food noise and hedonic drive (both elements of automatic motivation), EOMs may allow reflective motivation to exert greater influence over food choice. This shift does not assume that EOMs will change what people value; rather, the hypothesis is that reduced automatic drive may make it easier for individuals to act in ways consistent with their existing priorities. We frame this Perspective as hypothesis-generating: in triangulating converging but indirect evidence we hope articulate a framework that might provoke and guide further inquiry.

## Quieting the Food Noise: The Foundation for a New Dietary Paradigm

The capacity of EOMs to quiet food noise and reshape eating behavior is rooted in their profound physiological efficacy. EOMs induce reported energy intake reductions of 16% to 39% [32] to facilitate weight loss in the range of 15% to 22% [33,34], nearing figures previously only accessible through metabolic surgery. As of November 2025, it is estimated that 12% of adults are currently using EOMs, and 18% report ever use [35]. Even with the rapid uptake over the previous 3 y, the market demand far outstrips current access because of a host of factors such as cost [36], limited awareness/knowledge [37], lack of or inconsistent insurance coverage [38], currently available effective modalities (e.g., lower acceptability of injections compared with oral formulations) [37], and healthcare provider stigma [39].

Although such projections are inherently uncertain, several converging trends—competitive pressures, expanding EOM pipelines poised to commoditize older effective variants [40], more acceptable modalities supporting easier administration [41], and other potential evolutions (e.g., Medicare coverage)—make it plausible that a substantially larger share of adults with overweight and obesity will have consistent access to EOMs within the next 5 to 10 y. This projection is strengthened by emerging research suggesting myriad health benefits independent of weight loss, opening the possibility of indications for EOMs extending across diverse domains such as cardiovascular [42], renal [43], hepatic [44], neurodegenerative [45], and even behavioral reward domains unrelated to food (e.g., compulsive shopping) [46].

This potential marked uptake of EOM use stands to change eating patterns, and possibly improve dietary quality, in ways previously thought improbable given the sobering history of adherence to dietary recommendations. The profound efficacy by which EOMs reduce ad libitum energy intake is owed to mechanisms beyond simple appetite suppression. Although early attention was paid predominantly to physiological sensations such as nausea (which can lead to food aversion) and slowed gastric emptying (which can lead to satiation), it is increasingly apparent that the weight-lowering effects are likely more attributable to modulating hedonic and homeostatic control of eating [47]. EOMs are uniquely described as turning down the food noise to such a degree that focused efforts are increasingly made to operationalize and map future research directions in this domain [14,15]. The full extent and range of responses to various EOMs remains an intensely complex topic that is only beginning to be elucidated; pharmacologic profiles vary across agents (Box 1) and there is currently a limited evidence base to disentangle molecule-specific from class-wide effects. However, some commonalities or themes are being uncovered; themes which may facilitate a long-advised shift toward plant-forward dietary patterns.

### Cue-driven eating and hedonic friction to plant-forward patterning

Emerging experimental work supports the notion that EOMs attenuate cue-driven eating in ways that could lower the hedonic friction of an overall healthful dietary pattern more focused on plant foods. In this study, “friction” refers to the combined tradeoffs and decision burden that might make healthier defaults harder to execute in the moment. This plant-forward shift is theoretically possible because across diverse designs, EOMs reduce responsivity within key reward and salience pathways which translate into less cue-driven eating [29,[48], [49], [50]]. One recently published randomized controlled trial (RCT) assessed the early adaptive changes in eating behavior over 6 wk in response to tirzepatide therapy (Box 1) relative to liraglutide and placebo [50]. Consistent with previous research [51,52], tirzepatide reduced ad libitum energy intake when provided lunch (−534 ± 134 kcal compared with placebo) at the primary week 3 assessment, amounting to a 59% reduction in energy intake at lunch compared with baseline. In parallel, there were reductions in fasting appetite, hunger, and prospective consumption, all without an increase in cognitive restraint, suggesting less volitional restriction and a more automatic dampening of drive. Notably, tirzepatide lowered Food Craving Inventory overall scores, wherein cravings across all categories decreased—including high-fat foods, sweets, carbohydrates, starches or fast-food fats—with the singular exception of fruits and vegetables. Convergent measures showed reduced state cravings, lower responsiveness to the food environment, and modest reductions in impulsiveness. Functional magnetic resonance imaging demonstrated reduced activation to high-fat/high-sugar food images in regions mediating reward/salience at week 3 compared with placebo, consistent with selective dampening of cue-reactivity to palatable foods rather than a blanket suppression of all food motivation. Together, these data suggest that EOMs reduce “wanting” for energy-dense, high-fat/high-sugar foods while sparing motivation for fruits and vegetables, potentially enabling lower-friction adoption of a plant-forward pattern. In COM-B terms [31], this selective attenuation of wanting represents a dampening of automatic motivation for energy-dense foods, while leaving reflective motivation (the conscious desire for health- or values-aligned eating) relatively elevated.

### Meal state and late-meal effects favor plant-forward eating

A key concept that must be addressed to understand gradations of EOM-related eating behavior modulation stems from the distinctiveness of “wanting” and “liking” as constructs. Wanting—the motivation to pursue a reward—is quite distinct from liking—the enjoyment one gets from that reward [53,54]. We can want what we do not like, and like (enjoy) what we do not want (pursue). Crucially, both can operate independently of reflective motivation (conscious goals and values). Although EOMs plausibly affect both domains, it is increasingly apparent that “wanting” is more strongly modulated than liking, with modest or no changes reported in liking during consumption [55]. This is quite unique, as the reverse is true in typical weight-loss scenarios—“liking” decreases with weight loss >5%, whereas “wanting” is maintained [56].

Meal state further dissociates “liking” and “wanting.” In the satiated state, participants “liked but did not want” high-fat savory foods and, conversely, “wanted” low-fat sweet items more than they liked them—an effect which was amplified after a savory preload (i.e., a late-meal shift toward sweet) [57,58]. These findings suggest that interventions which sustain a fed-state signal—such as EOMs—could selectively attenuate “wanting” for savory/fatty foods while leaving other categories less affected. Indeed, these findings were bolstered by a 12-wk RCT whereby an EOM (once-weekly semaglutide) lowered total ad libitum energy intake by ∼24% and specifically reduced intake from high-fat, nonsweet items by ∼35% [51]. Collectively, these provocative findings suggest, but do not yet prove that “end-of-meal” preferences may persist throughout the entirety of EOM.

### Preference-intake dissociations with unhealthful foods

A 2024 United States consumer survey of current, potential, and nonusers of EOMs suggest another dimension by which EOMs can plausibly predispose individuals to a plant-forward shift: preference-intake dissociations [59]. Researchers document that while on EOMs, the largest reductions occur in the consumption of processed foods, sugar-sweetened beverages, refined grains, and beef. Conversely, the only food categories that saw a net increase were fruits and leafy greens [59]. Another recent analysis of EOM users found similar increases in fruit and vegetable intake [60]. These findings are largely in agreement with consumer purchasing data reflective of spending reductions across snack foods, meat, eggs, and bread [61]. However, the most intriguing finding may be that despite the decreased consumption of processed foods, sugar-sweetened beverages, refined grains, and beef, desirability for these foods was high and remained unchanged [59]. Study authors highlight this contradiction, “Generally, food preferences and consumption go side-by-side. However, despite having a higher desirability, EOM users decreased consumption of high-calorie foods.” This points to a unique dissociation between preference and consumption, adding another layer of complexity to the liking-wanting constructs previously discussed. This dissociation may be related to the greater perceived control without increased cognitive constraint reported by EOM users, allowing them to reduce consumption of certain foods without undue influence from elements that may contribute to food noise.

### Cognitive bandwidth freed for subordinated values

As automatic motivation (hedonic drivers such as taste and convenience) weakens its grip on food choice, the influence of reflective motivation (conscious goals, values, and identity) may be relatively amplified. Values do not change; rather, their expression becomes less obstructed. EOMs stand to enhance the capacity for longer-term value consideration by reducing the cognitive load associated with managing immediate appetitive drives. Accordingly, patients report greater alignment with physiological cues (eating only when hungry or “physically necessary”) and less emotion- or boredom-driven eating [49], creating the cognitive “space” to act in line with higher-order goals. The fundamental value structure likely remains intact (individuals do not necessarily develop new values) but the relative influence of existing values seems to shift substantially. This recalibration theoretically would be strongest in decision contexts where multiple values are directly competing (e.g., choosing between a conventional animal product compared with a plant-based alternative with sustainability advantages).

## The Pharmacological Window: Habituating a Durable, Plant-Forward Eating Pattern

Although an EOM-facilitated plant-forward shift may produce broad health [62] and environmental [3,63] benefits, the benefits may be more specific and profound for those seeking to maintain weight loss. There are currently no evidence-based recommendations for long-term management of weight lost after cessation of EOM therapy [64]. The challenges of maintaining weight loss are well documented, and although not unique to EOM users and past users [65], merit discussion. The EOM discontinuation rates are often reported to exceed 50% within a year [66], with around two-thirds of prior lost weight being regained within 1 y after discontinuation [67]. Although these sobering data rightfully fuel calls for better access and insurance coverage, we must also pragmatically recognize that for many, EOMs will not be a lifelong therapy (or at least not without some degree of usage fluctuation). This reality invites a strategic reframing whereby EOMs can open a “pharmacological window” to building lasting, self-sustaining habits.

Why call it a “window?” The most dramatic neurobehavioral shifts in response to EOMs appear early and may attenuate with time. For instance, one trial found EOMs reduced central responses to food cues at ∼10 d, but this effect was no longer detectable at 12 wk [29]. Likewise, reductions in reward-region activation observed at week 3 were already diminished by week 6 in a recent EOM trial [50]. In other words, EOMs seem to be lowering the volume of food noise, but the strongest dampening of cue-reactivity appears to be front-loaded. The temporal contours of this window remain provisional, and it is unclear whether this duration is sufficient for durable habit consolidation. This waning of suppression of food noise argues for a proactive strategy, one where RDNs are involved from the outset to help patients capitalize on this early period when automatic motivation may be most pliable.

By (temporarily) dampening food noise through the mechanisms previously outlined, individuals stand to gain a greater perceived degree of behavioral control which permits higher-order values (e.g., animal welfare, sustainability, health, etc.) to guide choices more consistently. This “window” is permissive of alignment of diet with personal ideals which can habituate to a plant-forward pattern while pharmacotherapy attenuates short-term reward conflicts. If habituated, identity-anchored dietary habits may be more durable; although it must be noted that this proposition draws on evidence from nonpharmacologic dietary contexts and remains to be tested under EOM therapy specifically [68,69]. When dietary choices affirm a salient identity (ethical, environmental, and religious), adherence tends to be stronger relative to other motives, such as weight motives [68]. Indeed, social-identification and self-efficacy are the strongest predictors of dietary adherence, whereas motivation by mood or weight control is associated with worsened adherence [69].

The identity hinge corresponds with a point raised in recent theorizing on food noise: “… the most effective diet to promote weight change is typically unclear. Restricting the diet for moral or religious reasons, on the other hand, provides a clear guideline for food choice” [14]. Food noise may thrive in environments with an overabundance of options and uncertainty while diets with clear, internalized guardrails reduce ambiguity and the moment-to-moment need to navigate tradeoffs. Pharmacologically turning down food noise may therefore make identity-consistent defaults easier to adopt, which may persist even as pharmacological effects fade.

The pharmacological window’s potential to facilitate a plant-forward pattern likely varies by individual and can be understood through the lens of reflective motivation. We propose 3 likely response patterns: First, individuals whose reflective motivation already aligns with plant-forward eating (e.g., through environmental values, ethical commitments, etc.) may find that EOMs lessen the automatic barriers that previously confounded their dietary intentions. For these individuals, the pharmacological window represents liberation rather than transformation. Second, individuals with strong identity-based opposition to plant-forward eating (e.g., following a “carnivore” pattern) are unlikely to shift toward plant-forward patterns regardless of EOM effects on automatic drive. Third, and perhaps most prevalent, are individuals whose reflective motivation favors “eating healthier” in general terms but who make suboptimal choices because of automatic hedonic responses and environmental cues. For this group, EOMs may create the conditions to align behavior with pre-existing health and lifestyle goals, without requiring identity transformation or value change.

Weight maintenance, rather than initial loss, may be where a plant-forward pattern offers the greatest advantage. Short-term success can be found with a variety of eating patterns [70,71], and in the EOM era, much of the initial energy deficit might be pharmacologically supported. There is likely no specific advantage of more plant-forward patterns toward eliciting an energy deficit and weight loss [72]. However, characteristics common to plant-forward patterns might be more conducive to longer-term weight stability. Accordingly, higher scores on the plant-based diet index were associated with better 3-y weight-loss maintenance in one trial [73], mirroring findings from a systematic review of prospective cohort studies [74]. These findings are representative of the broader weight maintenance literature: what supports weight loss may not be what supports weight-loss maintenance, given the dichotomous nature by which there is only a partial overlap of drivers and mechanisms [75].

Dietary patterns that are both identity-congruent and compatible with a given environment tend to be the ones sustained [69]. For many, which will mean pragmatically approaching a 1:1 plant-to-animal protein ratio rather than vegetarianism; for some, it will mean leaning further into plant-forward principles in ways that feel value-consistent. For example, a plant-based analog might replace a familiar animal dish for one person because it aligns with sustainability goals [76]; another might keep seafood and low-fat dairy but shift the “center of the plate” toward legumes and vegetables. Although there are numerous routes to achieving a more plant-forward pattern, diet modeling in older adults indicates that the plant-to-animal protein ratio can shift upward from the current 1:2 to 2:1 before nutrient adequacy becomes an issue, so long as legume intake increases and sufficient intakes of seafood and dairy remain [77]. These adequacy considerations may apply with particular force to dietary protein during EOM-induced energy restriction, where the relative contributions of protein are more apparent [78]. Although recent evidence suggests that lean mass changes during EOM therapy are broadly comparable with those observed with other weight-loss modalities [79,80], a plant-forward shift under reduced total intake warrants more explicit attention to protein nutrition [81]. With a plant-forward shift, the emphasis is on feasibility, and any movement from the current approximately two-thirds animal protein norm [23] might be achievable with modest changes.

None of these EOM-diminished frictions absolves many capability or opportunity gaps to behavior change [31]. EOMs do little to address notable barriers to plant-forward diets including food access, cost, time scarcity, or culinary skills [82]; these barriers persist and require support from RDNs, as well as broader policy solutions. EOMs do not directly alter the food environment (opportunity), but diminished automatic responses may functionally expand the range of dietary choices individuals perceive as accessible. Conversely, EOMs most directly open the window through strong modulation of automatic motivation—the behavior change lever underlying some of the most salient barriers such as craving/temptations and habit/automaticity [82]—which has so often overwhelmed adherence to dietary guidance to date. Thus, by fundamentally altering the psychological context of food choice, EOMs quiet the noise that has drowned out decades of dietary guidance. They may create a temporary window where the friction to adopt a healthier diet is lower than ever before. The ultimate success of this new era may not be measured by the weight lost while on these medications, but by the durable, identity-affirming habits that remain long after.

## Author contributions

The authors’ responsibilities were as follows – REB: led the review of the literature, wrote the primary draft of the manuscript; AWB, MJM, MMB: contributed domain expertise, drafted elements of the manuscript, and critically reviewed content; and all authors: read and approved the final manuscript.

## Data availability

Not applicable; this Perspective presents no new data.

## Funding

This work was funded by the Soy Nutrition Institute Global with support from the United Soybean Board.

## Declaration of generative AI and AI-assisted technologies in the manuscript preparation process

The authors declare that no generative AI or AI-assisted technologies were used in the writing of this manuscript.

## Conflicts of interest

REB reports a relationship with Soy Nutrition Institute Global that includes consulting or advisory. REB reports a relationship with Nlumn that includes consulting or advisory. REB reports a relationship with UnDun Longevity Innovations that includes consulting or advisory. MJM and MMB are employees of Soy Nutrition Institute Global which received funding from the United Soybean Board. In the 3 y before submission, AWB has received travel expenses from Alliance for Potato Research and Education, Institute for the Advancement of Food and Nutrition Sciences, International Food Information Council Foundation, National Academies of Science, Engineering, and Medicine, Novo Nordisk Foundation through the Understanding Processed Foods and Their Health Effects (UPDATE) project, Soy Nutrition Institute Global, and Toronto 3D Knowledge Synthesis and Clinical Trials Foundation; speaking honoraria from Alliance for Potato Research and Education, American Society for Nutrition, Eat Well Global, and National Restaurant Association; editorial board payments from American Society for Nutrition Statistical Review Board, and Springer Publishing Company (International Journal of Obesity); consulting payments from International Food Information Council Foundation, and Soy Nutrition Institute Global; and grants through his institution from American Egg Board, National Pork Board, NIH, and National Science Foundation. He has served as an unremunerated Board of Trustee member for the International Food Information Council. He has been involved in research for which his institution or colleagues have received grants or contracts from Alliance for Potato Research & Education, Childhood Arthritis and Rheumatology Research Alliance, National Pork Board, and NIH. His wife has been employed by Mead Johnson.
